# Improving Thermal, Mechanical, and Barrier Properties of Feather Keratin/Polyvinyl Alcohol/Tris(hydroxymethyl)aminomethane Nanocomposite Films by Incorporating Sodium Montmorillonite and TiO_2_

**DOI:** 10.3390/nano9020298

**Published:** 2019-02-20

**Authors:** Shufang Wu, Xunjun Chen, Minghao Yi, Jianfang Ge, Guoqiang Yin, Xinming Li, Ming He

**Affiliations:** 1 Green Chemical Engineering Institute, Zhongkai University of Agriculture and Engineering, Guangzhou 510225, China; SFWu2018@163.com (S.W.); MHYi0848@163.com (M.Y.); ge650704@163.com (J.G.); yingq007@163.com (G.Y.); lixinming@sina.com (X.L.); heming1026@163.com (M.H.); 2 Guangzhou Key Laboratory for Efficient Utilization of Agricultural Chemicals, Guangzhou 510225, China

**Keywords:** feather keratin, tris(hydroxymethyl)aminomethane, sodium montmorillonite, TiO_2_, solvent casting, nanocomposite

## Abstract

In this study, feather keratin/polyvinyl alcohol/tris(hydroxymethyl)aminomethane (FK/PVA/Tris) bionanocomposite films containing two types of nanoparticles, namely one-dimensional sodium montmorillonite (MMT) clay platelets (0.5, 1, 3, and 5 wt%) and three-dimensional TiO_2_ nanospheres (0.5, 1, 3, and 5 wt%), are prepared using solvent casting method. X-ray diffraction studies confirm the completely exfoliated structure of FK/PVA/Tris/MMT nanocomposites. The successful formation of new hydrogen bonds between the hydroxyl groups of the film matrix and the nanofillers is confirmed by Fourier transform infrared spectroscopy. The tensile strength, elongation at break, and initial degradation temperature of the films are enhanced after MMT and TiO_2_ incorporation. The water vapor permeability, oxygen permeability, and light transmittance decrease with increase in TiO_2_ and MMT contents. In summary, nanoblending is an effective method to promote the application of FK/PVA/Tris blend films in the packaging field.

## 1. Introduction

Environmental pollution and shortage of resources have forced researchers to explore biodegradable packaging materials as a replacement for petroleum-based synthetic plastics [1]. However, biodegradable packaging has limitations in terms of heat resistance, water resistance, mechanical properties, and cost [2]. At the same time, the use of edible starch or protein to prepare packaging materials may cause competition for food resources. Nonfood-borne keratin is a promising candidate for biodegradable packaging.

Keratin is abundantly present in animal hair, feathers, and hooves (protein content 80–90%). In fact, feathers and hair are derivatives of keratinization of animal epidermal cells. It is estimated that the annual production of feathers, feather poles, and livestock slaughter (or its by-products) in the United States is approximately 3 to 4 billion tons [3] and that in China is more than 1.5 billion tons [4], most of which are treated as waste. This not only causes environmental pollution, but also wastage of resources. Keratin can be extracted from feathers by physical [5], chemical [6,7,8], or microbial methods [9,10], and keratin films can be prepared by casting [11,12,13], hot press forming [14], electrospinning [15,16,17], etc. By using keratin films in the packaging field, “white pollution” can be reduced and the added value of keratin can be increased.

The main disadvantage of biodegradable feather keratin (FK) film is that it is brittle and has poor toughness (in the absence of plasticizer). These properties can generally be improved by blending and cross-linking. From our previous research, we found that blending of FK, polyvinyl alcohol (PVA), and tris(hydroxymethyl)aminomethane (Tris) can improve the mechanical properties of FK-based films [18]. However, the moisture sensitivity of the blend films is high, which limits their application in the packaging field. Cross-linking modification of FK-based film with CaCl_2_, transglutaminase, and genipin can improve its water resistance and mechanical properties; however, the barrier properties of cross-linked films are not good, especially oxygen barrier properties [19]. Therefore, other methods need to be explored to further improve the overall performance of FK-based films, especially mechanical, barrier, and thermal properties.

The use of nanomaterials to modify biodegradable polymers is an important approach to improving the properties of biodegradable polymers. Compared with traditional composite materials, a low percentage of nanomaterials can considerably increase the mechanical strength of the polymer, reduce weight, increase heat resistance, and improve the barrier properties of food packaging materials to moisture, oxygen, carbon dioxide, ultraviolet radiation, and volatiles. Montmorillonite is the most commonly used one-dimensional layered silicate material owing to its environmental friendliness and low-cost [20]. Abdallah et al. [21] used MMT which was prepared using spray freeze-drying (SFD-MMT) as reinforcement to prepare nanocomposites with poly(lactic acid) (PLA). It is shown that SFD-MMT provide satisfactory enhancements in rheological and mechanical properties of PLA. Song et al. [22] studied the effect of montmorillonite on the properties of keratin films. The results showed that the tensile strength of keratin nanocomposites increased with increase in the montmorillonite content (1–7%), and the water vapor permeability (WVP) reduced with increase in the montmorillonite content. Johannes Bott and Roland Franz [23] studied the migration potential of laponite in food. The authors claimed that the sample films with different loadings of laponite were stored for 10 days at 60 °C, and no migration of laponite was found at a limit of detection of 22 µg laponite per Kg food. It can be concluded that the laponite (which represents the worst case of any larger structural type of clay) does not migrate into the food once it is incorporated into the polymer matrix. 

Titanium dioxide (TiO_2_) has been the most widely studied three-dimensional nanoparticle in the past decade, because it is an inert and inexpensive material. TiO_2_ exhibits antibacterial properties and can be used for active packaging of foods [24]. It can also effectively inactivate antibiotic-resistant bacteria under ultraviolet irradiation [25], besides exhibiting photocatalytic activity. By blending the polymer with nano-TiO_2_, the physical properties of the polymer can be improved [26], biodegradability of the synthetic polymer can be increased [27,28], and degradation of organic pollutants can be promoted [29,30]. 

Considering the excellent properties of MMT and TiO_2_, nanoblending them with keratin will inevitably improve the physical performance of keratin films. To the best of our knowledge, studies on nanomodification of keratin with MMT and TiO_2_ are scarce. Therefore, in this study, MMT and TiO_2_ nanomodified FK/PVA/Tris blend films were prepared by the solution casting method, and the effects of their concentrations on the properties of FK/PVA/Tris blended films were investigated.

## 2. Materials and Methods

### 2.1. Materials

PVA (analytical grade, degree of polymerization = 1700, degree of alcoholysis = 99) was supplied by Aladdin Ltd. (Shanghai, China). Tris was provided by Shanghai Ebene Chemical Reagent Co., Ltd. (China). TiO_2_ (content = 99.8%, anatase) was purchased from Aladdin Ltd. (Shanghai, China). The average diameter of TiO_2_ particles (as reported by the company) is approximately 60 nm. Sodium montmorillonite (MMT, content > 98%), was obtained from Mingsen Plastic Materials Co. Ltd. (Dongguan, China), and chicken FK powder was prepared as described in our previous work [18]. Deionized (DI) water was employed as the solvent.

### 2.2. Preparation of 1% Nanoparticle Solutions

Solutions of MMT and TiO_2_ were prepared by adding MMT or TiO_2_ powder (1 g) and Tris (3 g) to DI water (96 g) at 30 °C with continuous stirring for 1 h, ultrasonication for 1 h (power ratio is 60%), and then continuous stirring for 24 h.

### 2.3. Preparation of the Nanocomposite Films

**Figure 1:**
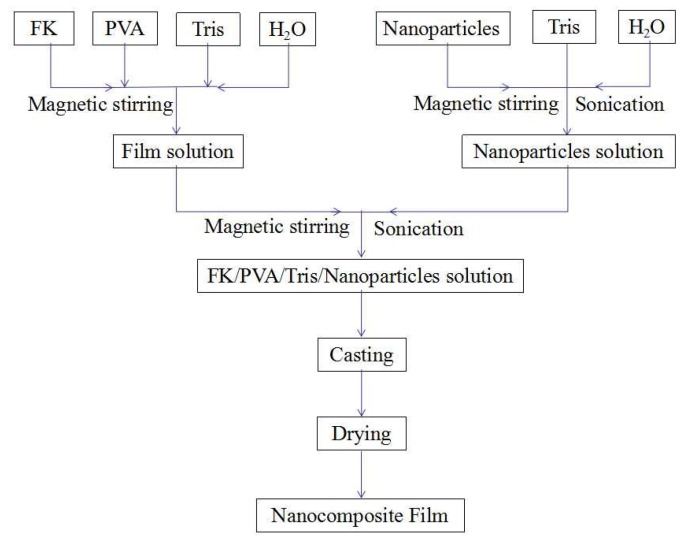
Different steps in the film forming process.

Referring to our previous work [18], P-40-25 (FK to PVA weight ratio of 60:40 and a Tris content of 25 wt% relative to the total weight of FK and PVA) was selected as the control group. The procedure for preparation of the nanocomposite films is as follows. The extracted FK powder (1.2 g) and 6% PVA solution (13.33 g) were weighed in a beaker (the total solid mass of FK + PVA was set to 2 g), and a certain amount of 6% Tris solution and DI water were added with continuous stirring at 40 °C for 30 min. Then, 1% of the nanoparticle solution consisting of 1, 2, 6, and 10 g (accounting for the total weight of FK and PVA 0.5%, 1%, 3% and 5%, respectively) was added with continuous stirring at 40 °C for 1 h. The specific amounts of each substance added are shown in Table 1. The total mass of the final film forming solution was maintained at 50 g. The solution was ultrasonicated for 0.5 h (power ratio of 60%), followed by magnetic stirring in a constant temperature water bath at 40 °C for 1 h. The cooled mixed solution was poured into polypropylene dishes (15 × 18 cm^2^). Nanomodified FK/PVA/Tris composite film was obtained by drying in a humidity chamber at 25 °C and 50% relative humidity for 24 h. The different steps in the film forming process are shown in Figure 1. The blend films were named x-MMT and x-TiO_2_, where x is the weight percentage of the nanoparticle relative to the total weight of FK and PVA in the film. The resulting blended films were conditioned at 25 °C and 50% relative humidity for 48 h prior to testing.

### 2.4. Characterization

The cross-sections (prepared by liquid nitrogen freezing) and the surface morphologies of the film samples were observed by scanning electron microscopy (SEM, EVO 18; Carl Zeiss, Jena, Germany) at an accelerating voltage of 10 kV to characterize the uniformity of nanoparticle distribution. Prior to the SEM observations, the samples were coated with a fine gold layer for 45 s.

Fourier transform infrared spectroscopy (FTIR) studies of the film samples were carried out using an infrared spectrometer (Spectrum 100, Perkin-Elmer, Fremont, CA, USA) in the attenuated total reflectance mode between 4000 and 650 cm^−1^ (eight scans per wavenumber). The MMT and the TiO_2_ powders were tested between 4000 and 450 cm^−1^ (four scans per wavenumber) by tableting with potassium bromide.

The crystallinity of the sample was determined by powder X-ray diffraction (XRD, D-MAX 2200 VPC, RIGAKU Company Ltd., Tokyo, Japan) with Cu Kα irradiation at an applied voltage of 40 kV (30 mA), 2θ scan range of 3 to 80°, and a scan speed of 5°·min^−1^.

Thermogravimetric analysis (TGA) was carried out using a thermogravimetric analyzer (TGA2, Mettler Toledo, Switzerland), and the mass of the sample tested was 3–5 mg. Under the protection of N_2_, the gas flow rate was maintained at 50 ml·min^−1^. The temperature range of the test was 40 to 700 °C, and the heating rate was 10 °C·min^−1^. 

The tensile properties of the film samples were measured using a microcomputer-controlled electronic universal testing machine (CMT6503, Shenzhen MTS Test Machine Company Ltd., Shenzhen, China), according to the ASTM D 882 standard, at a speed of 10 mm·min^−1^ and a fixture distance of 40 mm. The films were cut into samples measuring 75 mm × 10 mm, and the sample thicknesses were measured using a digital external micrometer (accurate to 0.001 mm). The measurements were conducted in triplicate and average values calculated.

The WVP values of the films were measured using a water vapor transmittance tester (W3/030, Labthink Ltd., Jinan, China). First, all the film samples were cut into circular shapes with diameters of 3 cm and were filled into the instrument. Then, the test conditions were set as follows; temperature: 38 °C, relative humidity across the film: 90–0%, warm-up time: 4 h, and weighing interval: 2 h. The measurements were conducted in triplicate and average values calculated.

The oxygen permeability (OP) values of the films were measured using an oxygen permeability tester (VAC-VBS, Labthink Ltd., Jinan, China) according to the GB/T 1038-2000 standard. Firstly, the samples were cut into circular shapes with diameters of 5.5 cm and placed in the instrument. Then, the test conditions were set as test gas pressure: 1.01 × 10^5^ Pa and upper and lower degassing time: 4 h. The measurements were conducted in triplicate and average values calculated.

The transmittance of the films in the wavelength range from 200 to 800 nm was measured using a spectrophotometer (UV-1800, Shimadzu Corporation, Chengdu, China) according to the method described by He et al. [13]. The film sample was cut into rectangular pieces (10 × 40 mm^2^) and directly attached to the cuvette. Empty cuvettes were used as blank controls. The transparency value (T) of the films was calculated using the following equation.

$$T=-(\log T_{600})/x$$

where T_600_ is the transmittance at 600 nm and *x* is the film thickness (mm). The measurements were conducted in triplicate and average values calculated.

## 3. Results and Discussion

### 3.1. Morphology of the Nanocomposite Films

Upon visual observation, all the nanocomposite films exhibited smooth surfaces with no obvious pores (see Supplementary Materials Image S1). The appearance of the MMT nanocomposite films was similar to that of the control film P-40-25: yellowish in color and optically transparent. However, with the addition of TiO_2_, the films gradually whitened and the light transmittance decreased. This is because TiO_2_ is a white coloring agent and has a strong scattering effect on light [26,31].

**Figure 2:**
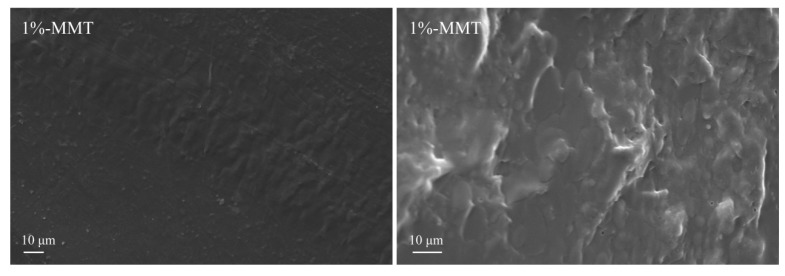

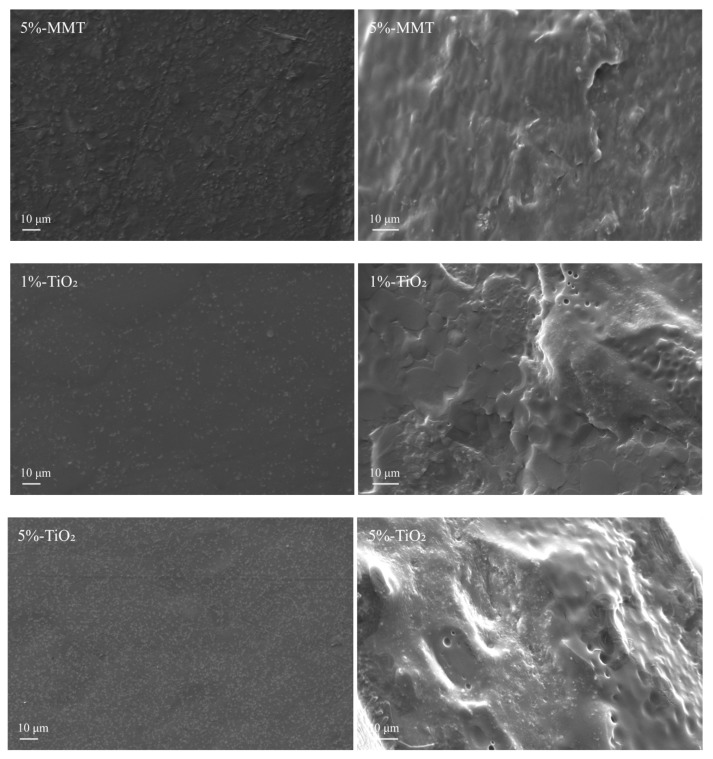
Representative SEM images of the surface morphology (left; 2000X) and the fracture morphology (right; 3000X) of the blend films.

To elucidate the influence of the addition of nanomaterials on the microstructure and the uniformity of the blend films, the surface and the cross-section of 1%-MMT, 5%-MMT, 1%-TiO_2_, and 5%-TiO_2_ nanocomposite films were analyzed by SEM, and the results are shown in Figure 2. All the blend films exhibit a rough surface with granular protrusions, and more granules are observed in the film matrix with increase in the content of MMT and TiO_2_. These granular protrusions are evenly distributed on the surface of the blend film, and no large aggregates of particles were observed, indicating that the nanoparticles are uniformly dispersed in the film matrix. The analysis of the cross-section can better indicate the dispersion uniformity of the nanoparticles in the blend film. As evident from the cross-sectional structure of the blend films, the addition of nanoparticles roughened the cross-section, and small particles are embedded in the film matrix. With increase in the content of nanoparticles, more particles are observed along the side of the cross-section (e.g., left side of the cross-sectional SEM image of 5%-TiO_2_ sample), which may be attributed to the deposition of nanoparticles during the film formation process. The more the amount of nanoparticles added, the more significant is the deposition, resulting in uneven distribution of the nanoparticles in the cross-section of the blend film.

### 3.2. FTIR Analysis

**Figure 3:**
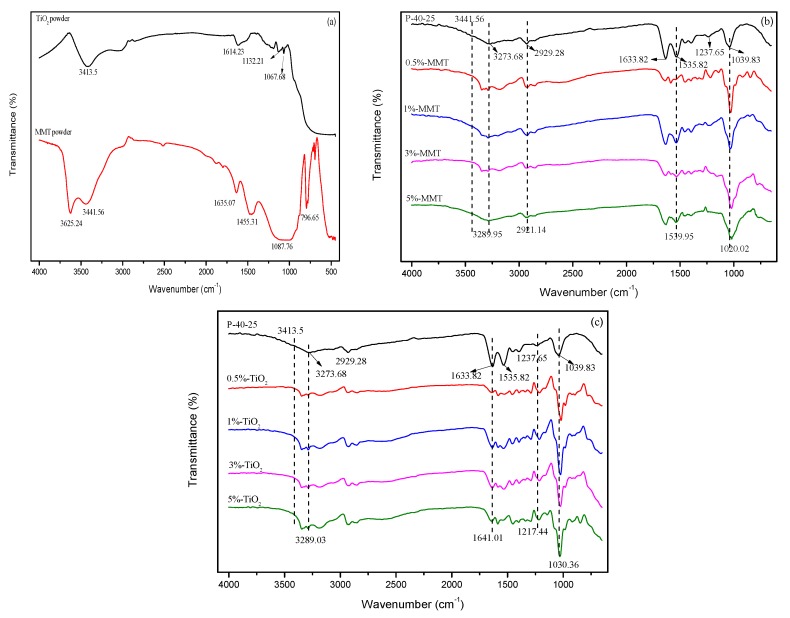
FTIR spectra of (**a**) MMT powder and TiO_2_ powder, (**b**) P-40-25 incorporated with MMT, and (**c**) P-40-25 incorporated with TiO_2_.

Figure 3a shows the FTIR spectra of pure MMT and TiO_2_ in the wavelength range of 4000–450 cm^−1^. In the case of MMT, the peaks observed at 3625.24, 3441.56, and 1087.76 cm^−1^ correspond to the MMT surface-OH stretching vibration, MMT interlayer-OH stretching vibration, and silicon bond in MMT structure [32], respectively. In the FTIR spectrum of TiO_2_, the peaks at 3413.5, 1614.23, and 1000–450 cm^−1^ correspond to -OH stretching vibration of TiO_2_ surface, -OH bending vibration, and Ti-O-Ti stretching vibration [33], respectively.

To fully understand the interaction between the FK/PVA/Tris blend film and the nanoparticles, the FTIR spectra of different nanomodified FK/PVA/Tris blend films were investigated (Figure 3b,c). The infrared spectra of the blend films changed significantly after nanomodification and the peak positions shifted. When 5%-MMT was added to the blend film, the FTIR peaks of the control film P-40-25 [19] at 3327.68, 2929.28, 1535.82, and 1039.83 cm^−1^ (corresponding to O–H and N–H association, –CH stretching vibration, amide II band, and C–O stretching vibration, respectively) shifted to 3289.95, 2921.14, 1539.95, and 1021.83 cm^−1^, respectively. At the same time, the absorption peaks of MMT at 3625.24 and 3441.56 cm^−1^ disappeared; this is because MMT-dispersed films exhibited exfoliated nanomorphology (as confirmed using XRD), and the hydroxyl groups between the interlayer and the surface of the layer were affected. When 5%-TiO_2_ was added to the blend film, the absorption peaks of P-40-25 [19] at 3327.68, 1633.82, 1237.65, and 1039.83 cm^−1^ (corresponding to O–H and N–H association, amide I band, amide III band, and C–O stretching vibration) shifted to 3289.03, 16410.11, 1217.44, and 1030.36 cm^−1^, respectively, and the absorption peak of TiO_2_ at 3413.5 cm^−1^ disappeared. These changes in the FTIR spectrum indicate the formation of strong hydrogen bonding between the nanoparticles and the film matrix [26].

### 3.3. XRD Studies

**Figure 4:**
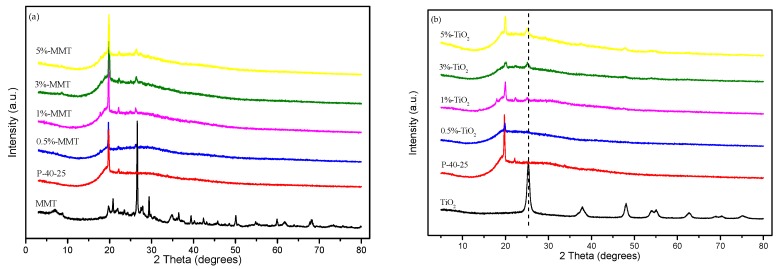
XRD patterns of (**a**) MMT: P-40-25, 0.5%-MMT, 1%-MMT, 3%-MMT, and 5%-MMT and (**b**) TiO_2_: P-40-25, 0.5%- TiO_2_, 1%- TiO_2_, 3%- TiO_2_, and 5%- TiO_2_.

To study the dispersion properties of the nanoparticles in the film matrix, XRD analyses of pure MMT, TiO_2_ powders, and blend films were carried out. Figure 4a shows the XRD pattern of MMT powder and FK/PVA/Tris nanocomposite films incorporated with different concentrations of MMT. For pure MMT, the characteristic diffraction peak appears at 2θ = 7.07°, and the layer spacing corresponding to d_001_, as calculated using Bragg’s law, is 1.249 nm [34]. For the MMT nanocomposite film, the peak at 7.07° disappeared. This result indicates an exfoliated nanomorphology, suggesting that the molecular chains of FK, PVA, and Tris entered into the gallery space of the silicate layers. Exfoliation and dispersion of MMT nanoplatelets may be due to strong interactions between FK, PVA, Tris, and MMT [34], as confirmed by FTIR studies. The hydroxyl groups of FK, PVA, and Tris can directly interact with the sodium ions of the MMT gallery (similar to water molecules) or with the edge hydroxyl groups of the MMT layers, and the amino groups of FK and Tris can interact with the hydroxyl groups of the MMT surface, forming a very compatible system. Ali et al. [35] reported that MMT (addition amount < 10%) was present in starch/PVA/MMT nanocomposite in an exfoliated nanomorphology. Similar results were observed in starch/MMT nanocomposites by Oleyaei et al. [26] (MMT additions were 3% or 5%); however, Song et al. [22] observed the presence of MMT in the intercalated structure of chicken feather protein/MMT composite film, even when the amount of MMT added was ≤ 7%.

Figure 4b shows the XRD pattern of TiO_2_ powder and FK/PVA/Tris nanocomposite film incorporated with different concentrations of TiO_2_. For pure TiO_2_ powder, the characteristic diffraction peaks appeared at 2θ = 25.32, 37.92, 48.08, 54.06, 55.14, 62.76, 68.8, 70.34, and 74.96°. It shows anatase characteristic structure with a lattice constant a = 3.785 Å and c = 9.514 Å, which is consistent with the anatase lattice TiO_2_ standard (JCPdS card nr 21-1272). With increasing the content of TiO_2_, the intensity of the anatase peak of 2θ = 25.32° in the blend film increased, and the peak position shifted to the low wave number and moved to 25.1° of 5%-TiO_2_. If there is no interaction between TiO_2_ and the film matrix, a simple superposition according to the ratio of these materials can be observed in the XRD pattern of the quaternary nanocomposite film, but the peak intensity of the quaternary nanocomposite changes and the peak position moved, it can be judged that there is an interaction force between them, which is consistent with the FTIR spectrum analysis.

Similar X-ray diffraction patterns were observed in all nanocomposite films with different MMT or TiO_2_ concentrations, since the main substrates of the film were FK, PVA, and Tris. At the same time, it can be seen that the addition of nanomaterials leads to a decrease in the peak intensity of the crystallization peak near 20° and a broadening of the peak width, indicating that the crystallization performance of the blend film is degraded, which may be due to the nanomaterial. The strong interaction between the nanomaterial and the film matrix reduced the degree of crystallinity of the blend films. 

### 3.4. TGA

**Figure 5:**
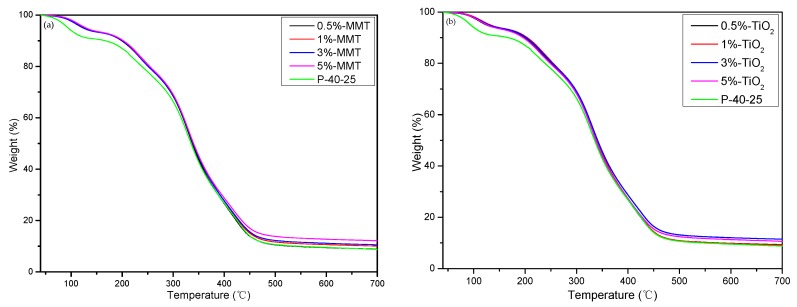
Thermogravimetric curves of (**a**) P-40-25 incorporated with MMT and (**b**) P-40-25 incorporated with TiO_2_.

The thermal degradation behavior of the samples was evaluated using TGA. Figure 5 shows that all the blend films exhibit two stages of weight loss: the first stage (Δ1) occurs between 40 and 150 °C, mainly because of loss of free water and combined water [36] and the second stage (Δ2) occurs between 200 and 500 °C, which is mainly related to the degradation behavior of the mixture, i.e., accompanied by decomposition during the melting process [37]. The temperatures of the different degradation stages and the final residual amounts are listed in Table 2; T_d1_ is the temperature at which the mass loss is 10%, T_onset_ is the initial degradation temperature in the second stage, and T_max_ is the fastest degradation temperature in the second stage (the peak value of first derivative of TG curve). In the first stage, when the mass loss is 10%, the T_d1_ of the nanocomposite film is approximately 30 °C higher than that of P-40-25. This can be attributed to the barrier effect of the nanomaterial, which can form a protective physical barrier on the surface of the material, thereby hindering the evaporation of water vapor from the blend films. Thus, the nanocomposite films can reach the same amount of water vapor evaporation loss at a higher temperature. In the second stage, the T_onset_ of the nanocomposite film was significantly improved, compared with that of P-40-25; the increase was ~14–21 °C. In addition, T_max_ improved slightly, and the final residual amount increased. These data indicate that the addition of nanoparticles can improve the thermal stability of the FK/PVA/Tris blend film owing to the high thermal stability of the nanoparticles and the barrier effect of the nanoparticles. Nanoparticles can form a protective physical barrier on the surface of the material, which prevents volatile degradation products from escaping from the composite and ultimately retards the degradation of the composite [38,39].

### 3.5. Mechanical Properties

**Figure 6:**
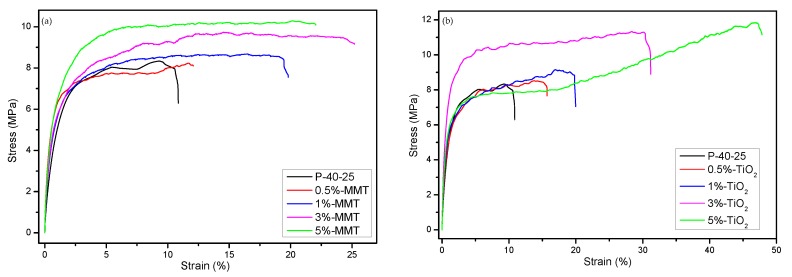
Tensile properties of (**a**) P-40-25 incorporated with MMT and (**b**) P-40-25 incorporated with TiO_2_.

**Figure 7:**
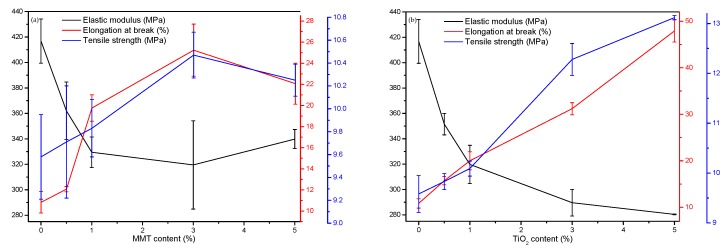
Tensile properties of (**a**) P-40-25 incorporated with MMT and (**b**) P-40-25 incorporated with TiO_2_.

Figure 6 shows the stress–strain relationship of the control film and the nanocomposite films. It is evident that the addition of nanoparticles improves the mechanical properties, i.e., both the elongation at break and the tensile strength of the FK/PVA/Tris blend films. Figure 7 shows the elastic modulus, tensile strength, and elongation at break of the FK/PVA/Tris blend films prepared with different contents of MMT and TiO_2_. Tensile properties for P-40-25 were from Chen et al. [18]. The specific mechanical properties of the nanocomposite films can be found in the Supplementary Materials (Table S1). The elastic modulus of P-40-25 reduced from 416.78 MPa to 339.85 (5%-MMT) and 280.38 MPa (5%-TiO_2_); the elongation at break of P-40-25 increased from 10.83% to 22.07 (5%-MMT) and 47.9% (5%-TiO_2_); and the tensile strength of P-40-25 increased from 9.58 MPa to 10.25 (5%-MMT) and 13.12 MPa (5%-TiO_2_). It is worth noting that the two different nanoparticles imparted different effects on the mechanical properties of the FK/PVA/Tris blend films, and these mechanical properties varied at different concentrations of the various nanoparticles. 

In the MMT nanocomposite film (Figure 7a), the elongation at break and the tensile strength of the MMT nanoblend film first increased and then decreased with increase in the MMT content, whereas the elastic modulus first decreased and then increased. This is attributed to the physical adsorption and hydrogen bonding force between the MMT and the film matrix. When the MMT content is ≤3%, MMT is uniformly dispersed into the film matrix owing to the exfoliated structure, and the interfacial interaction between the film matrix and MMT enhances with increase in the MMT content. This results in better transfer of load or force from the film matrix to the nanoclay filler, while not easily breaking the film matrix–nanoclay interface during tensile deformation. With increase in the MMT content to 5%, MMT begins to aggregate, reducing the interfacial interaction between the film matrix and the nanoclay filler, thereby resulting in interfacial failure and decrease in the tensile strength and the elongation at break. Similar results were observed in tilapia skin gelatin/MMT blend films by Nagarajan et al. [40]. 

In the case of TiO_2_ nanocomposite film (Figure 7b), the elongation at break and the tensile strength of the TiO_2_ nanoblend film increased with increase in the TiO_2_ content, and the elastic modulus decreased. A possible reason is that when the film substrate is subjected to an external force, it is easy to excite the surrounding matrix to generate microcracks and absorb energy because of the stress concentration effect of the rigid TiO_2_ nanoparticles. At the same time, the existence of rigid particles hinders and inactivates crack propagation in the matrix, preventing destructive cracking, thereby resulting in enhanced toughness [41]. Another reason is that TiO_2_ can strengthen the association of O–Ti–O bonds between the film matrix and the nanoparticles through electrostatic attraction and hydrogen bonding, thereby forming a stable three-dimensional polymer matrix and improving the elongation and the tensile strength of the blend films [31,42].

Comparing the data in Figure 7, at the same content of nanoparticles, the mechanical properties of the TiO_2_ nanocomposite films are better than those of the MMT nanocomposite films. Thus, nano-TiO_2_ is more effective than MMT in improving the mechanical properties of the blend films. This may be related to the compatibility and the dispersion uniformity of the nanoparticles with the film matrix. The water solubility of TiO_2_ is better than that of MMT, and the dispersion of TiO_2_ in the film matrix is more uniform; hence, the mechanical properties of the TiO_2_ nanocomposite films are better than those of MMT nanocomposite films at the same concentration of nanoparticles.

### 3.6. Barrier Properties of the Blend Films

Barrier performance is a measure of the resistance of packaging materials to the scent of the package as well as intrusion of gases, bacteria, etc. present outside the package. Materials with good barrier properties ensure the stability of the packaged product and extend its shelf life. Therefore, the barrier properties of packaging materials seriously affect their use in the field of packaging. The following sections describe the WVP, oxygen permeability, and UV barrier properties of the blend films.

#### 3.6.1. WVP

**Figure 8:**
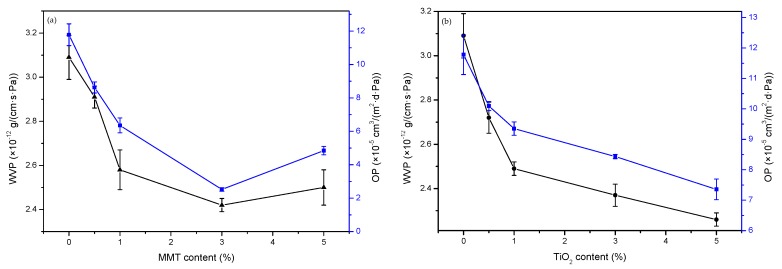
Water vapor permeability (WVP) and oxygen permeability (OP) values of (**a**) P-40-25 incorporated with MMT and (**b**) P-40-25 incorporated with TiO_2_.

The effects of the two nanoparticles on the WVP values of P-40-25 are shown in Figure 8. Figure 8a shows the WVP values of P-40-25 incorporated with different contents of MMT. With increase in the MMT content, the WVP value of the films decreased first and then increased slightly. When the MMT content is 3%, the WVP of the nanocomposite film reaches a minimum of 2.42 × 10^−12^ g·cm^−1^·s^−1^·Pa^−1^, which is 21.68% lower than that of P-40-25 [18]. This may be attributed to the strong interaction of MMT with the film matrix, which reduces the free volume of the film matrix and interferes with the transport of water vapor through the film matrix, extending the path of water vapor diffusion through the film matrix [43]. In addition, Li et al. [44] pointed out that MMT as a physical cross-linking site can enhance the stability of the network. The strong interaction between the film matrix and the MMT sheet consumes some hydrophilic groups, which decreases the water absorbed by capillary action at the interface, thereby hindering the diffusion of water molecules. When the MMT content was increased to 5%, the WVP of the blend film increased slightly, but it was still smaller than that of the control film. This may be caused by the accumulation of MMT in the film matrix, which affects the water vapor barrier performance of the composite film. Similar results have been reported in the literature; for example, the addition of MMT was found to improve the water vapor barrier properties of fish gelatin film [40,45], chicken feather protein film [22], and starch/PVA composite film [35].

Figure 8b shows the WVP values of P-40-25 incorporated with different contents of TiO_2_. The WVP value of the films decreased with increase in the TiO_2_ content. When the TiO_2_ content is 5%, the WVP of the nanocomposite film reaches a minimum of 2.26 × 10^−12^ g·cm^−1^·s^−1^·Pa^−1^, which is 26.86% lower than that of P-40-25 [18]. The possible reason is the lower water solubility and hydrophilicity of TiO_2_ compared with those of keratin, PVA, and Tris, which blocks micro- and nanopathways in network structures [26]. In addition, Zhou et al. [42] have reported that TiO_2_ has a hydrophobic coating effect on the surface of the film, which slows down the adsorption rate and reduces the WVP.

Therefore, the water vapor barrier properties of the blend films of MMT and TiO_2_ nanoparticles are better than that of the control film. In particular, the water vapor resistance of TiO_2_ blend film is superior to that of MMT blend film.

#### 3.6.2. Oxygen Permeability

The oxygen permeability (OP) values of P-40-25 incorporated with different contents of MMT are also shown in Figure 8a. The OP value of the film decreased first and then increased slightly with increase in the MMT content, a trend consistent with that of WVP. When the MMT content is 3%, the OP of the nanocomposite film reaches a minimum of 2.521 × 10^−5^ cm^3^·m^−2^·d^−1^·Pa^−1^, which is 78.6% lower than that of P-40-25 [18]. 

The OP value of P-40-25 incorporated with different contents of TiO_2_ is shown in Figure 8b. The oxygen barrier properties of the blend films decreased with increase in the TiO_2_ content. When the TiO_2_ content is 5%, the OP of the nanocomposite film reaches a minimum of 7.355 × 10^−5^ cm^3^·m^−2^·d^−1^·Pa^−1^, which is 37.56% lower than that of P-40-25 [18]. 

The addition of MMT and TiO_2_ nanoparticles improves the oxygen barrier properties of the blend films because of the strong interaction between the nanoparticles and the film matrix; small-sized nanoparticles have a higher occupation of the porous film matrix. The filling up of the voids reduces the free volume of the film matrix and interferes with the transport of oxygen through the film matrix, extending the path of diffusion of oxygen through the film matrix, thereby reducing the amount of oxygen permeation [46].

#### 3.6.3. Light Transmission and Transparency

Table 3 shows the values of transmittance of the MMT and the TiO_2_ nanomodified FK/PVA/Tris blend films at selected wavelengths in the range of 200 to 800 nm. With the addition of nanoparticles, the transmittance of the blend film decreased at all wavelengths, and the degree of reduction varied depending on the type and the content of the nanoparticles. When the content of nanoparticles is the same, the blend film with TiO_2_ has a smaller transmittance than that with MMT, indicating that the TiO_2_ nanocomposite film has higher barrier light transmission performance than the MMT nanocomposite film. This is because TiO_2_ is a semiconductor and absorbs photon energy to excite electrons from the valence band to the conduction band, resulting in enhanced absorbance of the TiO_2_ film and reduced light transmission [47]. Meanwhile, the light transmittance of the FK/PVA/Tris blend film decreased with increase in the nanoparticle content, which may be caused by light scattering effect of the nanoparticles distributed in the blended film matrix. It is worth noting that the addition of TiO_2_ reduced the transmittance of the blended film in the visible light region (800 nm) from 79.65% in P-40-25 to 2% in 5%-TiO_2_ nanocomposite, and the transmittance in the ultraviolet region (350 nm) reduced from 18.4% in P-40-25 to 0% in 5%-TiO_2_ nanocomposite. In addition, the transmittance varied significantly with the concentration of TiO_2_. Similar results have been reported with the addition of TiO_2_ in starch [26,48], whey protein [42], and kefir–whey protein complex [49]. The light transmittance of all the films is less (T < 1%) in the ultraviolet region (wavelengths below 300 nm), indicating that the blend film has good UV resistance. Therefore, the addition of MMT and TiO_2_ can improve the UV-barrier properties of the FK/PVA/Tris blend film.

**Figure 9:**
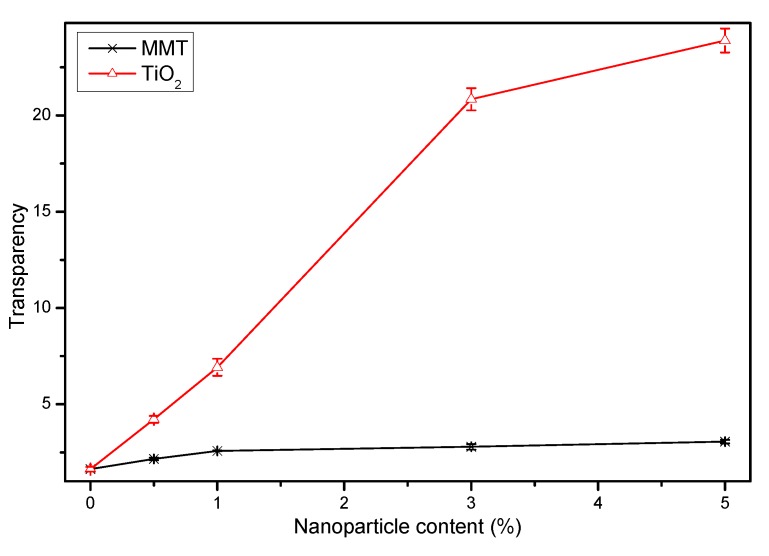
Transparency of the FK/PVA/Tris blend films modified with different contents of MMT and TiO_2_.

It can be seen from the transparency values in Figure 9 that the transparency value of the nanocomposite film is higher than that of P-40-25, and the transparency value of the film increases with increase in the content of nanoparticles. The specific transparency values of the blend films can be found in the Supplementary Materials (Table S2). At the same content of nanoparticles, the film containing TiO_2_ exhibits higher transparency value than that containing MMT. The higher the transparency value, the lower is the transparency of the film and vice versa. Thus, the transparency of P-40-25 is better than that of the nanocomposite blend film, and the transparency of MMT-modified FK/PVA/Tris blend film is better than that of TiO_2_-modified blend film. The transparency of a film is generally affected by additives, processing conditions, thickness, and compatibility between the polymer and the nanoparticle [50,51,52,53,54]. The limited compatibility between the nanoparticles and the matrix of FK/PVA/Tris, especially with increase in the nanoparticle content, may result in agglomeration of the nanoparticles, increasing the internal scattering of light and the turbidity [55]. Therefore, the addition of nanoparticles has an effect on the appearance and the photoresistance properties of the FK/PVA/Tris blend film, depending on the type of nanoparticles and the level of addition.

## 4. Conclusions

In this study, we successfully prepared FK/PVA/Tris nanocomposite films containing MMT or TiO_2_. XRD studies showed that silicate platelets were exfoliated and TiO_2_ nanoparticles were uniformly dispersed in the film matrix. The shifting of the characteristic peaks of P-40-25, MMT, and TiO_2_ nanoparticles in the FTIR spectra indicated the formation of new hydrogen bonds in the nanocomposite matrix. It was observed that FK/PVA/Tris blend films containing MMT or TiO_2_ exhibited improved thermal, mechanical, and barrier properties compared with the unfilled material. Specifically, P-40-25 loaded with 3% of MMT exhibited maximum improvement in the properties: the elongation at break and the tensile strength increased by 133.06% and 9.29%, and the WVP and the OP decreased by 21.68% and 78.6% when compared to those of P-40-25. Meanwhile, P-40-25 loaded with 5% TiO_2_ exhibited maximum improvement in the properties: the elongation at break and the tensile strength increased by 434.6% and 36.95%, and the WVP and the OP decreased by 26.86% and 37.56% when compared to those of P-40-25. It is worth noting that the addition of MMT and TiO_2_ can improve the UV resistance of the films. Furthermore, the MMT nanocomposite films retained the visible transparency, while the TiO_2_ nanocomposite films increased the opacity. This study provides a method to improve the properties of keratin films for packaging applications.

## Figures and Tables

**Table 1:** Compositions of the different samples of blend films.

Sample	FK Powder (g)	6% PVA (g)	6% Tris (g)	1% MMT (g)	1% TiO_2_ (g)	H_2_O (g)
P-40-25	1.2	13.33	8.33	0	0	27.17
0.5%-MMT	1.2	13.33	7.83	1	0	26.67
1%-MMT	1.2	13.33	7.33	2	0	26.17
3%-MMT	1.2	13.33	5.33	6	0	24.17
5%-MMT	1.2	13.33	3.33	10	0	22.17
0.5%-TiO_2_	1.2	13.33	7.83	0	1	26.67
1%-TiO_2_	1.2	13.33	7.33	0	2	26.17
3%-TiO_2_	1.2	13.33	5.33	0	6	24.17
5%-TiO_2_	1.2	13.33	3.33	0	10	22.17

**Table 2:** Thermogravimetric analysis (TGA) results of P-40-25 incorporated with MMT and TiO_2_.

Sample	Δ1	Δ2		Residue (%)
	T_d1_ (°C)	T_onset_ (°C)	T_max_ (°C)	
P-40-25	167.5	214.83	329.33	8.75
0.5%-MMT	199.17	231.83	330.67	8.86
1%-MMT	202.17	235.83	330.83	10.07
3%-MMT	200.5	230.17	330.17	10.55
5%-MMT	202.33	230	329.67	12.11
0.5%-TiO_2_	199.67	229.67	330.17	9.29
1%-TiO_2_	204	234.5	331	9.23
3%-TiO_2_	201.83	236.5	331.33	11.44
5%-TiO_2_	196.17	227.5	330	10.64

**Table 3:** Transmittance of the FK/PVA/Tris blend films modified with different contents of MMT and TiO_2_.

Sample	%T								
	800 nm	700 nm	600 nm	500 nm	400 nm	350 nm	300 nm	280 nm	200 nm
P-40-25	79.65	77.57	74.21	66.73	41.49	18.40	0.67	0.10	0.00
0.5%-MMT	74.10	71.22	67.10	59.56	37.83	18.21	0.62	0.04	0.00
1%-MMT	68.55	65.65	61.95	54.25	32.92	18.05	0.53	0.00	0.00
3%-MMT	67.60	63.20	59.40	51.20	27.30	16.40	0.38	0.00	0.00
5%-MMT	62.30	60.00	56.19	47.80	25.10	14.50	0.20	0.00	0.00
0.5%-TiO_2_	56.15	51.81	45.91	37.64	21.95	11.46	0.10	0.00	0.00
1%-TiO_2_	41.00	35.30	27.80	20.50	9.20	3.80	0.10	0.00	0.00
3%-TiO_2_	6.00	3.70	2.10	1.30	0.50	0.00	0.00	0.00	0.00
5%-TiO_2_	2.00	1.40	1.10	0.80	0.30	0.00	0.00	0.00	0.00

## Supplementary Materials

The supplementary materials are available online at http://www.mdpi.com/2079-4991/9/2/298/s1.
