# Supplementary material for: Improving Thermal, Mechanical, and Barrier Properties of Feather Keratin/Polyvinyl Alcohol/Tris(hydroxymethyl)aminomethane Nanocomposite Films by Incorporating Sodium Montmorillonite and TiO2
Source: Nanomaterials (Basel). 2019 Feb 20;9(2):298. doi: 10.3390/nano9020298 (PMC6409987; doi:10.3390/nano9020298)
Supplement: Supplementary file 1 [file nanomaterials-09-00298-s001.pdf]

Data article

Macroscopic appearance, thickness, mechanical data, transmittance and transparency data and contact angle data of nano-blend films.

Authors: Shufang Wu

Affiliations: Zhongkai University of Agriculture and Engineering.

Contact email: SFWu2018@163.com

Abstract

Macroscopic photos, thickness, mechanical data, transmittance and transparency data and contact angle data of nano-blend films can be seen in this data article .

Specifications Table *[please fill in right-hand column of the table below]*

Subject area	*Material*
More specific subject area	*Nanomaterials*
Type of data	*image, table, figure*
How data was acquired	*Macroscopic appearance (telephone,Huawei FIG-AL 10, China) , thickness (digital external micrometer 727II-01, accurate to 0.001 mm, China), tensile properties analysis (microcomputer-controlled electronic universal testing machine CMT6503, China), transmittance and transparency data (spectrophotometer UV-1800, China), contact angle data (automatic contact angle meter Theta, Finland))*
Data format	*Raw, analyzed*
Experimental factors	*The pretreatments of samples can be found in the experimental section below.*
Experimental features	*The various measurements can be found in the experimental section below.*
Data source location	*Guangzhou, China*
Data accessibility	*The data is with this article*

Value of the data

The macroscopic photos and thickness data of the blended films allows the researchers to visualize the look of the composite films. The mechanical data allows the researchers to understand the mechanical properties of the nano-modified blend films. Light transmission and transparency data allow researchers to understand the optical properties of nano-modified blend films. The contact angle data helps the researchers understand the changes in surface water resistance of the blended films after nano-modification.

Data

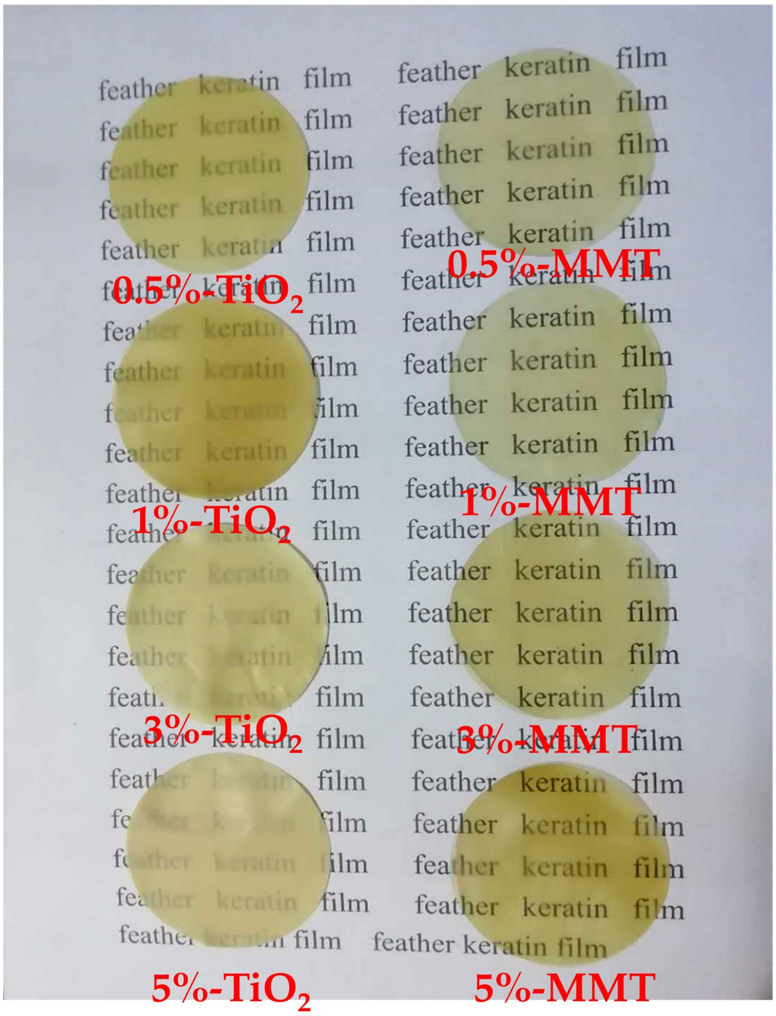

Image S1. *The Macroscopic photo of the blend films*

Table S1. *Tensile properties of the blend films*

Sample	Elastic modulus (MPa)	Elongation at break (%)	Tensile strength (MPa)	Thickness (mm)
P-40-25	416.78±17.34	10.83±1.01	9.58±0.37	0.087±0.004
0.5%-MMT	362.12±22.52	12.08±0.25	9.71±0.49	0.0848±0.007
1%-MMT	329.5±12.0	19.76±1.25	9.83±0.25	0.0883±0.008
3%-MMT	319.54±34.67	25.24±2.47	10.47±0.2	0.093±0.006
5%-MMT	339.85±7.52	22.07±1.94	10.25±0.14	0.1001±0.005
0.5%-TiO_2	351.56±8.48	15.71±0.88	9.83±0.16	0.0867±0.002
1%-TiO_2	319.78±15.1	20±1.94	10.09±0.15	0.0907±0.016
3%-TiO_2	289.59±10.51	31.19±1.3	12.28±0.32	0.0922±0.004
5%-TiO_2	280.38±0.28	47.9±2.38	13.12±0.04	0.0902±0.001

[1]Tensile properties for P-40-25 were from Chen et al. [1].

Table S2. *Transmittance and transparency of the FK/PVA/Tris blend films modified with different contents of MMT and TiO_2*

Sample	%T									Transparency
	800 nm	700 nm	600 nm	500 nm	400 nm	350 nm	300 nm	280 nm	200 nm	
P-40-25	79.65	77.57	74.21	66.73	41.49	18.40	0.67	0.10	0.00	1.63±0.12
0.5%-MMT	74.10	71.22	67.10	59.56	37.83	18.21	0.62	0.04	0.00	2.16±0.07
1%-MMT	68.55	65.65	61.95	54.25	32.92	18.05	0.53	0.00	0.00	2.58±0.02
3%-MMT	67.60	63.20	59.40	51.20	27.30	16.40	0.38	0.00	0.00	2.79±0.14
5%-MMT	62.30	60.00	56.19	47.80	25.10	14.50	0.20	0.00	0.00	3.06±0.09
0.5%-TiO_2	56.15	51.81	45.91	37.64	21.95	11.46	0.10	0.00	0.00	4.22±0.18
1%-TiO_2	41.00	35.30	27.80	20.50	9.20	3.80	0.10	0.00	0.00	6.92±0.44
3%-TiO_2	6.00	3.70	2.10	1.30	0.50	0.00	0.00	0.00	0.00	20.84±0.58
5%-TiO_2	2.00	1.40	1.10	0.80	0.30	0.00	0.00	0.00	0.00	23.89±0.62

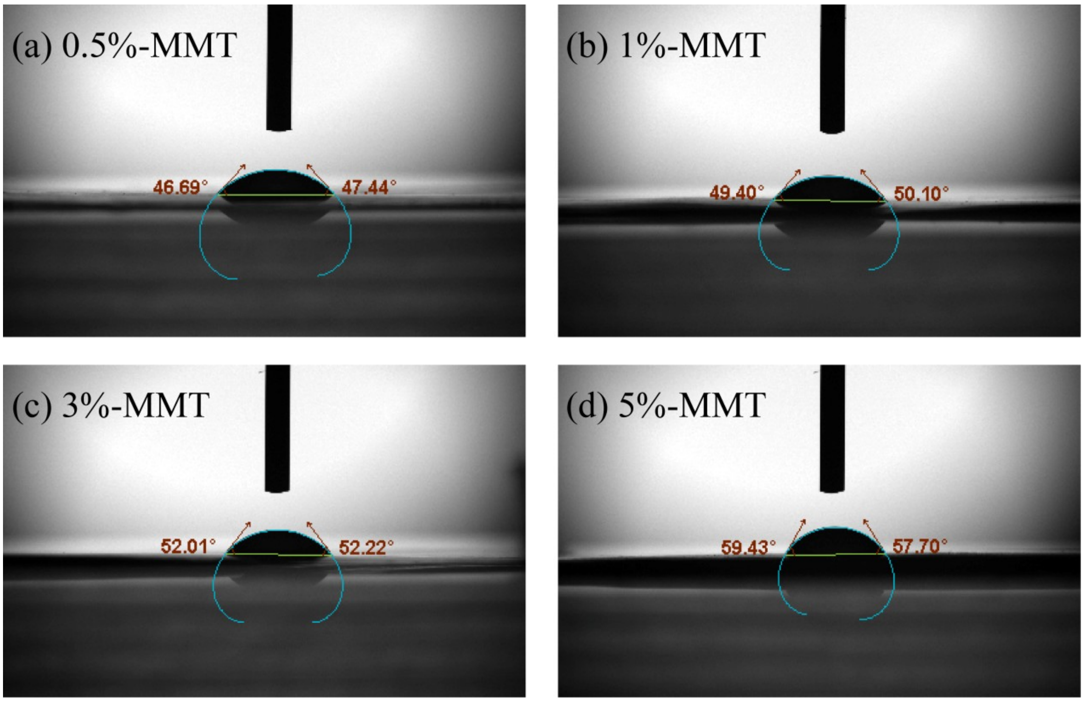

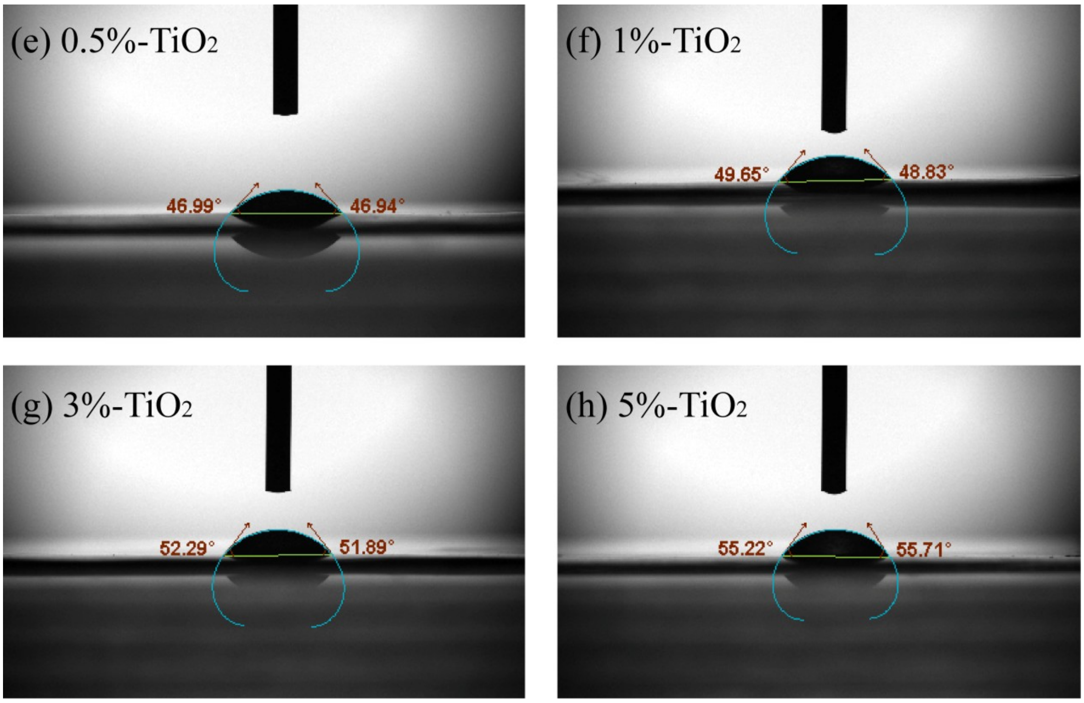

Figure S1. *The contact angles for the various blend films: (a) 0. 5%-MMT, (b) 1%-MMT, (c) 3%-MMT, (d) 5%-MMT, (e) 0. 5%-TiO_2, (f) 1%-TiO_2, (g) 3%-TiO_2, (h) 5%-TiO_2.*

Experimental Design, Materials and Methods

The tensile properties of the film samples were measured using a microcomputer-controlled electronic universal testing machine (CMT6503, Shenzhen MTS Test Machine Company Ltd., China), according to the ASTM D 882 standard, at a speed of 10 mm·min^{-1} and a fixture distance of 40 mm. The films were cut into samples measuring 75 mm × 10 mm, and the sample thicknesses were measured using a digital external micrometer (accurate to 0.001 mm). The measurements were conducted in triplicate and average values calculated.

The transmittance of the films in the wavelength range from 200 to 800 nm was measured using a spectrophotometer (UV-1800, Shimadzu Corporation, Chengdu, China) according to the method described by He et al. [2]. The film sample was cut into rectangular pieces (10 × 40 mm^2) and directly attached to the cuvette. Empty cuvettes were used as blank controls. The transparency value (T) of the films was calculated using the following equation.

$$T = -(\log T_{600})/\boldsymbol{x},$$

where T_{600} is the transmittance at 600 nm and x is the film thickness (mm). The measurements were conducted in triplicate and average values calculated.

Contact angle measurements were measured with an automatic contact angle meter (Theta, Biolin Scientific Ltd., Espoo, Finland). More specifically, the prepared filmsamples were attached to a glass slide, laid flat on a stage, and then deionized water droplets (3-7μL) were extruded from the needle tube

onto the sample surface. The contact angle was evaluated as the average of the measurements on both sides of the water droplet.

Acknowledgements
The authors thank the Analysis and Test Center at Zhongkai University of Agriculture and Engineering.

References

1. Chen, X.; Wu, S.; Yi, M.; Ge, J.; Yin, G.; Li, X. Preparation and physicochemical properties of blend films of feather keratin and poly(vinyl alcohol) compatibilized by tris(hydroxymethyl)aminomethane. *Polymers* **2018**, *10*, 1054-1065.
2. He, M.; Zhang, B.; Dou, Y.; Yin, G.; Cui, Y. Blend modification of feather keratin-based films using sodium alginate. *J. Appl. Polym. Sci.* **2017**, *134*, 44680-44687.
